# Benchmarks of Diabetes Care in Men Living With Treated HIV-Infection: A Tertiary Center Experience

**DOI:** 10.3389/fendo.2018.00634

**Published:** 2018-10-31

**Authors:** Monika Fazekas-Lavu, Katherine T. T. Tonks, Katherine Samaras

**Affiliations:** ^1^Department of Endocrinology, St Vincent's Hospital, Darlinghurst, NSW, Australia; ^2^Diabetes and Metabolism Program, Garvan Institute of Medical Research, Darlinghurst, NSW, Australia; ^3^St Vincent's Clinical School, University of New South Wales, Darlinghurst, NSW, Australia

**Keywords:** HIV and diabetes care, meeting benchmarked targets, comparison between HIV+DM and DM-alone, complication screening rates, are we meeting targets in both groups

## Abstract

Treated human immunodeficiency virus (HIV) infection is associated with high rates of type 2 diabetes mellitus (DM), metabolic syndrome and central obesity/body fat partitioning disorders. To our knowledge, there are no available data comparing diabetes care in people with both HIV+DM vs. DM alone (DM-controls) within the same service and evaluating if benchmarked standards of care are being met in people with HIV+DM. This study evaluated the frequency that people with HIV+DM met the benchmarked American Diabetes Association (ADA) standards of care in diabetes (targets for HbA1c, blood pressure, lipid levels, complication screening, and healthy weight), compared to age- and sex- matched controls with diabetes, in an urban teaching hospital. The frequency of diabetes complications and rates of obesity and metabolic syndrome were also examined. All participants were male; individuals with HIV+DM (*n* = 30) were similar to DM-controls (*n* = 30) for age, diabetes duration and smoking status, but were more frequently non-obese compared to DM controls (92 vs. 55%, respectively, *p* = 0.003). Only 41% of HIV+DM met HbA1c targets, compared with 70% of DM-controls (*p* = 0.037). Blood pressure targets were poorly met in both HIV+DM and DM-controls: 43 vs. 23%, respectively (*p* = 0.12); LDL cholesterol targets were met in 65 vs. 67% (*p* = 1.0). Benchmarked complication screening rates were similar between HIV+DM vs. DM-controls for annual foot examination (53 vs. 67%, respectively, *p* = 0.29); biennial retinal examination (83 vs. 77%, respectively, *p* = 0.52); and annual urinary albumin measurement (77 vs. 67%, respectively, *p* = 0.39). The prevalence of diabetes complications was similar between HIV+DM compared to DM-controls: macrovascular complications were present in 23% in both groups (*p* = 1.0); the prevalence of microvascular complications was 40 vs. 30%, respectively (*p* = 0.51). Achieving the standard of care benchmarks for diabetes in people with both HIV-infection and diabetes is of particular importance to mitigate against the accelerated cardiometabolic outcomes observed in those with treated HIV infection. HIV+DM were less likely to achieve HbA1c targets than people with diabetes, but without HIV. People with HIV+DM may require specific strategies to ensure care benchmarks are met.

## Introduction

According to the latest United Nations AIDS HIV data, there are currently 36.7 million people globally living with human immunodeficiency virus (HIV)-infection, with 19.5 million receiving combined anti-retroviral therapy (cART) (1). Advances in infectious disease detection and cART have increased life expectancy in people living with HIV-infection and acquired immunodeficiency syndrome (AIDS), with substantial diminution in the frequency of AIDS-related complications and deaths (2–6). As a result, there has been a rise in age-related conditions in this group, particularly cART-associated metabolic complications such as type 2 diabetes mellitus (DM) and cardiovascular disease, which are now major contributors to the increased morbidity and mortality associated with treated HIV-infection (2–6). Further, obesity (particularly central obesity) contributes to and accelerates cardiometabolic disease.

Diabetes is a serious health condition associated with increased morbidity and mortality. According to the World Health Organization 2016 global report on diabetes, there were an estimated 422 million adults living with diabetes in 2014, with an estimated global prevalence of 8.5%. Prevalence has almost doubled from the rate of 4.7% in 1980 (7). Risk factors include the global epidemic of obesity, superimposed on traditional risk factors such as family history, medications (including corticosteroids and combined antiretroviral therapy) and hepatitis C virus (HCV) infection (2).

The American Diabetes Association (ADA) publishes annual evidence-based diabetes standards of care practice guidelines to optimize the quality of care and health outcomes of people living with diabetes (8). The guidelines recommend a broad HbA1c target <7% (<53 mmol/mol), based on data demonstrating that better glycaemic control reduces incident microvascular complications and is associated with long-term reduction of incident macrovascular disease (8–10). Recommendations for blood pressure control are a systolic pressure <140 mmHg and diastolic pressure <80 mmHg, with lower readings in younger patients of <130/80 mmHg or even lower if comorbidities are present (8). The recommended lipid target is low-density lipoprotein (LDL) cholesterol <2.6 mmol/l (or <1.8 mmol/l in patients with history of cardiovascular disease), high-density lipoprotein (HDL) cholesterol >1.0 mmol/l in men and triglycerides <1.7 mmol/l (8). The ADA also recommends screening for complications with annual foot examination and urinary albumin excretion and biennial eye examination by an ophthalmologist or optometrist (8).

It is unclear how frequently people with HIV-infection and diabetes meet the benchmarked standards of diabetes care, whether complication rates are similar to people with diabetes without HIV, and whether treatment disparities exist for people living with both HIV-infection and diabetes.

The aims of this study were to evaluate (i) the frequency that diabetes care benchmarks were met in people living with treated HIV-infection and diabetes, including HbA1c levels and across the spectrum of cardiometabolic care and complication screening required in diabetes care, and (ii) whether a discrepancy exists in screening and meeting benchmarked targets for those with both HIV+DM compared to age- and sex- matched controls attending the same service.

## Materials and methods

Data were collected retrospectively from attendees at an Australian inner city tertiary referral teaching hospital. Patients known to be living with HIV-infection attending the hospital's ambulatory diabetes care services were identified. All were male. Therefore, inclusion criteria were males aged 18 years and over with both HIV+DM that attended at least one outpatient diabetes clinic over 12 months (January–December 2013). Once patients with both HIV+DM were identified, age-, and sex- matched controls were identified from diabetes clinic attendees who were not known to have HIV-infection that also attended the outpatient diabetes clinic between January and December 2013. Exclusion criteria were corticosteroid-induced diabetes, and transplant-related diabetes. Files were reviewed to collect data on age, weight, height, body mass index (BMI), smoking status, diabetes type, known diabetes duration, diabetes treatment, anti-hypertensive therapy, lipid lowering therapy, and HCV-co-infection (Table 1). In patients with HIV-infection, data were also collected on known HIV-infection duration, whether they were on cART, CD4 count, viral load and presence of AIDS-defining illnesses (Table 2).

**Table 1:** Demographic data of diabetes clinic attendees with HIV-infection and diabetes vs. diabetes controls.

	**HIV-infection and diabetes**	**Diabetes controls**	***p*-value**
Number of patients (*n*)	30	30	
Age (years)	56.7 ± 9.5	57.4 ± 10	0.78
BMI (kg/m^2^)	25.5 ± 3.1	28.9 ± 6.3	**0.021**
Smoker	5/23 (22%)	5/28 (18%)	0.73
**Patients with**			
T2DM	26/30 (87%)	30/30 (100%)	0.11
T1DM/LADA	4/30 (13%)	0/30 (0%)	0.11
Duration of DM (years)	9.6 ± 8.4	9.2 ± 6.3	0.83
**Diabetes therapy**			
- Metformin	21/30 (70%)	29/30 (97%)	**0.006**
- Sulfonylurea	14/30 (47%)	13/30 (43%)	0.80
- DDPIV/GLP1	3/30 (10%)	7/30 (23%)	0.17
- Insulin	11/30 (37%)	5/30 (17%)	0.08
**Antihypertensive therapy**			
ACEI/ARB	12/14 (86%)	21/21 (100%)	0.15
Other	5/11 (45%)	11/21 (52%)	0.71
**Lipid-lowering therapy**			
Statin	17/24 (71%)	18/21 (86%)	0.23
Fenofibrate	4/22 (18%)	2/21 (9.5%)	0.66
Co-infection w HCV	4/30 (13%)	1/30 (3.3%)	0.35

*Data expressed as mean ± SD OR as number (n) and Percentages. For continuous variables p-values were calculated with 2-sided independent T-test or Mann-Whitney test where data were non-parametric and for categorical variables with two sided Pearson Chi-square test or Fisher's exact 2-sided test if 2 or more cells with expected count <5; p < 0.05 considered significant. Bold values indicate statistically significant results (i.e., where p value is < 0.05)*.

**Table 2:** HIV-specific characteristics of diabetes clinic attendees with HIV-infection.

**HIV duration in years (*n* = 30)**	**19.3 ±7.3**
Number of patients on cART	29/30 (97%)
CD4 count (*n* = 28)	603 ± 262
Viral load detected?	5/27 (19%)
Viral load (*n* = 5)	8364 ± 14258
AIDS defining illness?^*^	5/30 (17%)

*cART, combined anti-retroviral therapy*.

* *AIDS defining illness–is the list of diseases published by the Centers for Disease Control and Prevention that are associated with AIDS and used worldwide as a guideline for AIDS diagnosis*.

The frequency of meeting diabetes care benchmarks was determined from the medical records, by evidence of measurement of blood pressure, foot examination, retinal examination, in addition to laboratory measures [HbA1c, lipids, urinary albumin to creatinine ratio (uACR)], number of diabetes clinic visits during the 12-month period, and whether patients had attended a dietician or diabetes educator (Table 3).

**Table 3:** Diabetes care benchmarks and prevalence of targets being met of diabetes clinic attendees with HIV-infection and diabetes vs. diabetes controls.

	**HIV-infection and diabetes**	**Diabetes controls**	***p*-value**
Mean HbA1c (%)	7.5 ± 1.5	7.0 ± 1.4	0.26
- Only type 2 diabetes (*n* = 25)	7.4 ± 1.4	7.0 ± 1.4	0.32
- HbA1c at target^*^	11/29 (41%)	21/30 (70%)	**0.037**
**Mean blood pressure**			
Systolic BP (mmHg)	126 ± 10	137 ± 17	**0.005**
Diastolic BP (mmHg)	77 ± 8	81 ± 9	0.08
- BP at target^**^	12/28 (43%)	6/26 (23%)	0.12
**Mean lipids**			
Total cholesterol (mmol/l)	5.0 ± 1.8	4.0 ± 1.0	**0.017**
HDL cholesterol (mmol/l)	1.1 ± 0.2	1.3 ± 0.4	0.07
- At target^***^	12/23 (52%)	17/24 (71%)	0.24
LDL cholesterol (mmol/l)	2.8 ± 2.9	2.0 ± 0.7	0.18
- At target^***^	13/20 (65%)	16/24 (67%)	1.0
Triglycerides (mmol/l)	3.6 ± 4.2	1.8 ± 1.3	**0.043**
- At target^***^	2/28 (7.1%)	4/26 (15%)	0.41
**Weight category by BMI**			
healthy (0–24.9)	10/26 (39%)	5/20 (25%)	**0.013**
overweight (25.0–29.9)	14/26 (54%)	6/20 (30%)	
Obese (30.0–100)	2/26 (7.7%)	9/20 (45%)	
**Weight category by BMI**			
Healthy (≤24.9)	10/26 (39%)	5/20 (25%)	0.33
Non-healthy (≥25.0)	16/26 (62%)	15/20 (75%)	
**Weight category by BMI**			
Non-obese (≤29.9)	24/26 (92%)	11/20 (55%)	**0.003**
Obese (≥30.0)	2/26 (7.7%)	9/20 (45%)	
Annual foot examination	16/30 (53%)	20/30 (67%)	0.29
Regular Podiatry visits	2/21 (10%)	6/22 (27%)	0.14
Bi-annual retinal examination	25/30 (83%)	23/30 (77%)	0.52
Annual urine albumin to creatinine measurement	23/30 (77%)	20/30 (67%)	0.39
**Visit # to diabetes clinic in 2013**			
1 visit	10/30 (33%)	15/30 (50%)	0.13
2 visits	13/30 (43%)	14/30 (47%)	
3 or more visits	7/30 (23%)	1/30 (3.3%)	
Seen a dietitian	17/30 (57%)	10/30 (33%)	0.07
Seen a diabetes educator	23/30 (77%)	14/30 (47%)	**0.017**

* *HbA1c target - T1DM < 8.0%; T2DM < 7.0%; T2DM with age >70 years or history of cardiovascular disease <7.5%*.

** *BP target < 130/80 mmHg or <125/75 mmHg if proteinuria w >1 g/d*.

*** *Lipid targets - LDL cholesterol <2.6 or <1.8 mmol/l if clinical macrovascular disease; HDL cholesterol >1.0 mmol/l; Triglycerides <1.7 mmol/l. Bold values indicate statistically significant results (i.e., where p value is <0.05)*.

ADA Standards of Care (2013) were used to define individualized targets (8): HbA1c <8.0% (64 mmol/mol) in type 1 diabetes mellitus (T1DM), HbA1c <7.0% (53 mmol/mol) in type 2 diabetes mellitus (T2DM) or HbA1c <7.5% (58 mmol/mol) in patients aged >70 years or with history of cardiovascular disease; LDL cholesterol level <2.6 and <1.8 mmol/l in patients with clinical macrovascular disease (i.e., history of cardiovascular disease and/or cerebrovascular disease); HDL cholesterol >1.0 mmol/l in men and triglyceride level <1.7 mmol/l. At the time of the study, the Australian Diabetes society mandated for even stricter hypertension targets with blood pressure (BP) targets <130/80 mmHg or <125/75 if proteinuria >1 g/day (11). These targets were used in our study. Weight status was determined by calculated body mass index (BMI; weight/squared height, kg/m^2^). Overweight was defined as BMI 25.0–29.9 kg/m^2^ and obese ≥30.0 kg/m^2^. Weight categories were further stratified into healthy BMI (≤24.9) vs. non-healthy BMI (≥25.0) and non-obese (≤29.9) vs. obese (≥30) (Table 3) (8).

The differences between HIV-infected individuals meeting and not meeting the HbA1c target (Table 4) and the differences between HIV-infected individuals with T1DM vs. T2DM (Table 5) were evaluated. To quantify the burden of diabetes complications, the presence of micro- and macro-vascular complications was documented (Table 6).

**Table 4:** Subgroup analysis of diabetes clinic attendees with HIV-infection and diabetes based on whether they met their HbA1c target^*^.

	HbA1c at target^*^	**HbA1c NOT at target**	***p*-value**
Number of patients (*n*)	12	17	
Age (years)	57.2 ± 7.7	57.2 ± 10.5	1.0
BMI (kg/m^2^)	24.3 ± 2.2	26.4 ± 3.4	0.07
Smoker	1/10 (10%)	4/13 (31%)	0.34
**Patients with**			
T2DM	10/12 (83%)	15/17 (88%)	1.0
T1DM/LADA	2/12 (17%)	2/17 (12%)	1.0
Duration of DM (years)	8.8 ± 7.8	10.6 ± 9.1	0.59
**Diabetes therapy**			
- Metformin	8/12 (67%)	12/17 (71%)	1.0
- Insulin	3/12 (25%)	8/17 (47%)	0.27
Mean systolic BP (mmHg)	124 ± 9.7	129 ± 9.4	0.48
Mean diastolic BP (mmHg)	78.3 ± 9.6	76.3 ± 6.6	0.52
Mean HDL cholesterol (mmol/l)	1.1 ± 0.3	1.1 ± 0.2	0.73
- At target^**^	3/8 (38%)	9/15 (60%)	0.40
Mean LDL cholesterol (mmol/l)	2.7 ± 0.9	2.0 ± 0.8	0.11
- At target^***^	3/6 (50%)	10/13 (77%)	0.32
Mean triglycerides (mmol/l)	3.2 ± 1.7	2.7 ± 2.8	0.61
- At target^****^	2/10 (20%)	6/17 (35%)	0.66
**Weight category by BMI (kg/m**^2^**)**			
Obese (BMI ≥30.0)	0/11	2/15 (13%)	0.49
**Weight category by BMI (kg/m**^2^**)**			
- Healthy (BMI ≤ 24.9)	7/11 (63%)	3/15 (20%)	**0.043**
- Non-healthy (BMI ≥25.0)	4/11 (36%)	12/15 (80%)	
HCV co-infection	1/12 (8%)	3/17 (18%)	0.62

* *HbA1c targets - T1DM < 8.0%; T2DM <7.0%; T2DM with age >70 years or history of cardiovascular disease—target <7.5%*.

** *HDL cholesterol target <1.0 mmol/l*.

*** *LDL cholesterol target <2.6 or <1.8 mmol/l if clinical macrovascular disease*.

**** *Triglycerides target <1.7 mmol/l. Bold values indicate statistically significant results (i.e., where p value is <0.05)*.

**Table 5:** Subgroup analysis of diabetes clinic attendees with HIV-infection and T1DM vs. T2DM.

	**HIV+T1DM**	**HIV+T2DM**	***p*-value**
Number of patients (*n*)	4	26	
Age (years)	47.0 ± 9.2	58.2 ± 8.8	**0.027**
BMI (kg/m^2^)	21.9 ± 3.0	26.2 ± 3.0	**0.007**
Smoker	1/2 (50%)	4/21 (19%)	0.40
Duration of DM (years)	22.5 ± 14.6	7.6 ± 5.2	0.59
**Diabetes therapy**			
- Metformin	0/4 (0%)	21/26 (81%)	**0.005**
- Insulin	4/4 (100%)	7/26 (27%)	**0.012**
Mean systolic BP (mmHg)	118 ± 3.0	127 ± 9.0	0.06
Mean diastolic BP (mmHg)	68.7 ± 9.0	78.6 ± 7.0	**0.018**
Mean HDL cholesterol (mmol/l)	1.15 ± 0.4	1.1 ± 0.2	0.69
Mean LDL cholesterol (mmol/l)	2.84 ± 1.1	2.81 ± 3.2	0.98
Mean triglycerides (mmol/l)	1.76 ± 0.7	3.85 ± 4.4	0.36
**Weight category by BMI (kg/m**^2^**)**			
Obese (BMI≥30.0)	0/4 (0%)	2/22 (9%)	1.00
**Weight category by BMI (kg/m**^2^**)**			
- Healthy (BMI ≤ 24.9)	3/4 (75%)	7/22 (32%)	0.26
- Non-healthy (BMI ≥25.0)	1/4 (25%)	15/22 (68%)	

*Bold values indicate statistically significant results (i.e., where p value is < 0.05)*.

**Table 6:** Macro and micro-vascular complications of diabetes clinic attendees wtih HIV-infection and diabetes vs. diabetes controls.

	**HIV-infection and diabetes**	**Diabetes controls**	***p*-value**
**Macro-vascular complications**			
Ischaemic heart disease	5/30 (17%)	5/30 (17%)	1.0
Cerebrovascular disease	3/30 (10%)	3/30 (10%)	1.0
Peripheral vascular disease	0/30 (0%)	0/30 (0%)	
**Micro-vascular complications**			
Diabetic retinopathy	2/30 (6.7%)	1/30 (3.3%)	0.55
Microalbuminuria or CKD	10/30 (33%)	5/30 (17%)	0.14
Peripheral neuropathy	7/30 (23%)	6/30 (20%)	0.75
Composite score for number of Macro-vascular Complications (0–3)	0–23/30 (77%)	0–23/30 (77%)	1.0
	1–6/30 (20%)	1–6/30 (20%)	
	2–1/30 (3.3%)	2–1/30 (3.3%)	
Composite score for number of Micro-vascular Complications (0–3)	0–18/30 (60%)	0–21/30 (70%)	0.51
	1–7/30 (23%)	1–6/30 (20%)	
	2–3/30 (10%)	2–3/30 (10%)	
	3–2/30 (6.7%)	3–0	

*CKD, chronic kidney disease*.

Data are expressed as means ± standard deviations, or number and percentage. Data analysis was performed with IBM SPSS, Version 24.0, Armonk, NY, USA, IBM Corp. Continuous variables were analyzed with Student's *t*-test or, where data were non-parametric, the Mann Whitney *U*-test. Categorical variables were analyzed with Chi-squared test or Fisher's 2-sided exact test. *P* < 0.05 was considered significant in statistical analyzes.

## Results

Table 1 shows the demographic data of the 60 male individuals. The groups were closely matched for age. Individuals with HIV+DM had lower BMI than with DM-controls (25.5 ± 3.1 vs. 28.9 ± 6.3 kg/m^2^, respectively, *p* = 0.021). The majority of HIV+DM had T2DM (*n* = 26, 87%); 4 had T1DM (13%). All of DM-controls had T2DM. Glucose lowering medications were similar for most medication classes, with the exception of metformin, which was less frequent in HIV+DM (70 vs. 97%, *p* = 0.006). Diabetes duration, use of lipid- and blood pressure- lowering medications were similar between HIV+DM and DM-controls. HCV-co-infection was more common in HIV+DM than DM-controls (13 vs. 3%, respectively), but not significant (Table 1).

For HIV+DM, the majority received cART (96%, *n* = 29); the known duration of HIV infection was 19.3 ± 7.3 years (Table 2). Other HIV-specific demographics are listed in Table 2.

### Meeting the ADA benchmarks

**Figure 1:**
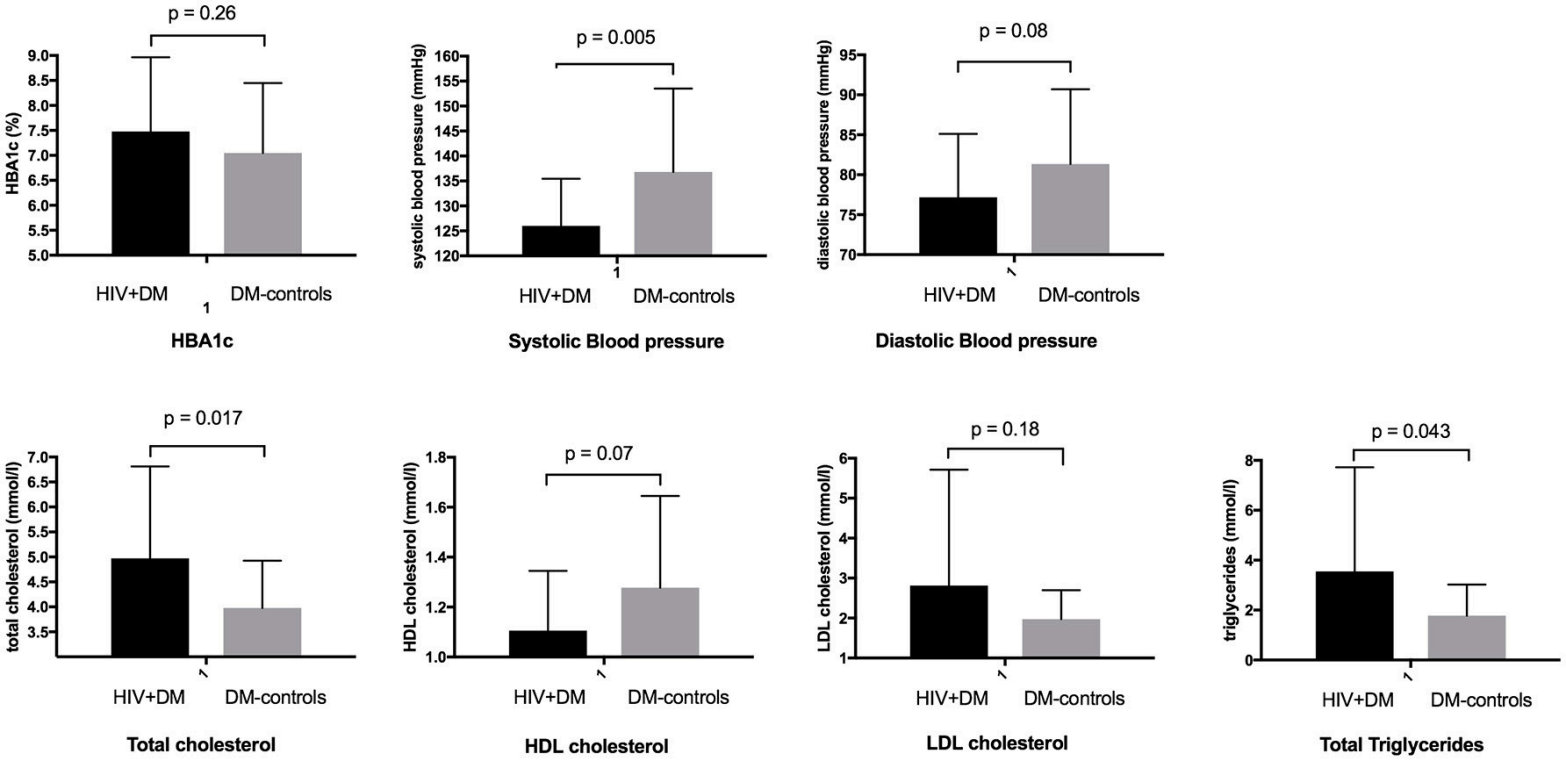
Diabetic care benchmarks in diabetes clinic attendees with HIV-infection compared to controls.

**Figure 2:**
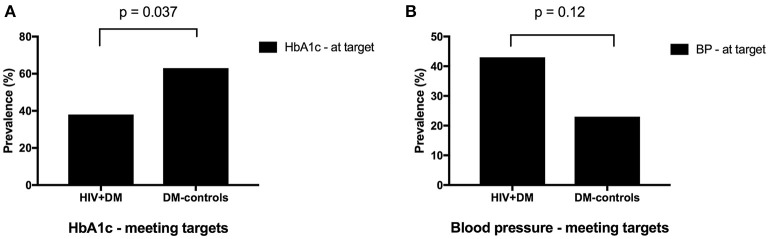
Proportion of clinic attendees meeting individualized ADA benchmarks for HbA1c **(A)** and blood pressure **(B)**.

**Figure 3:**
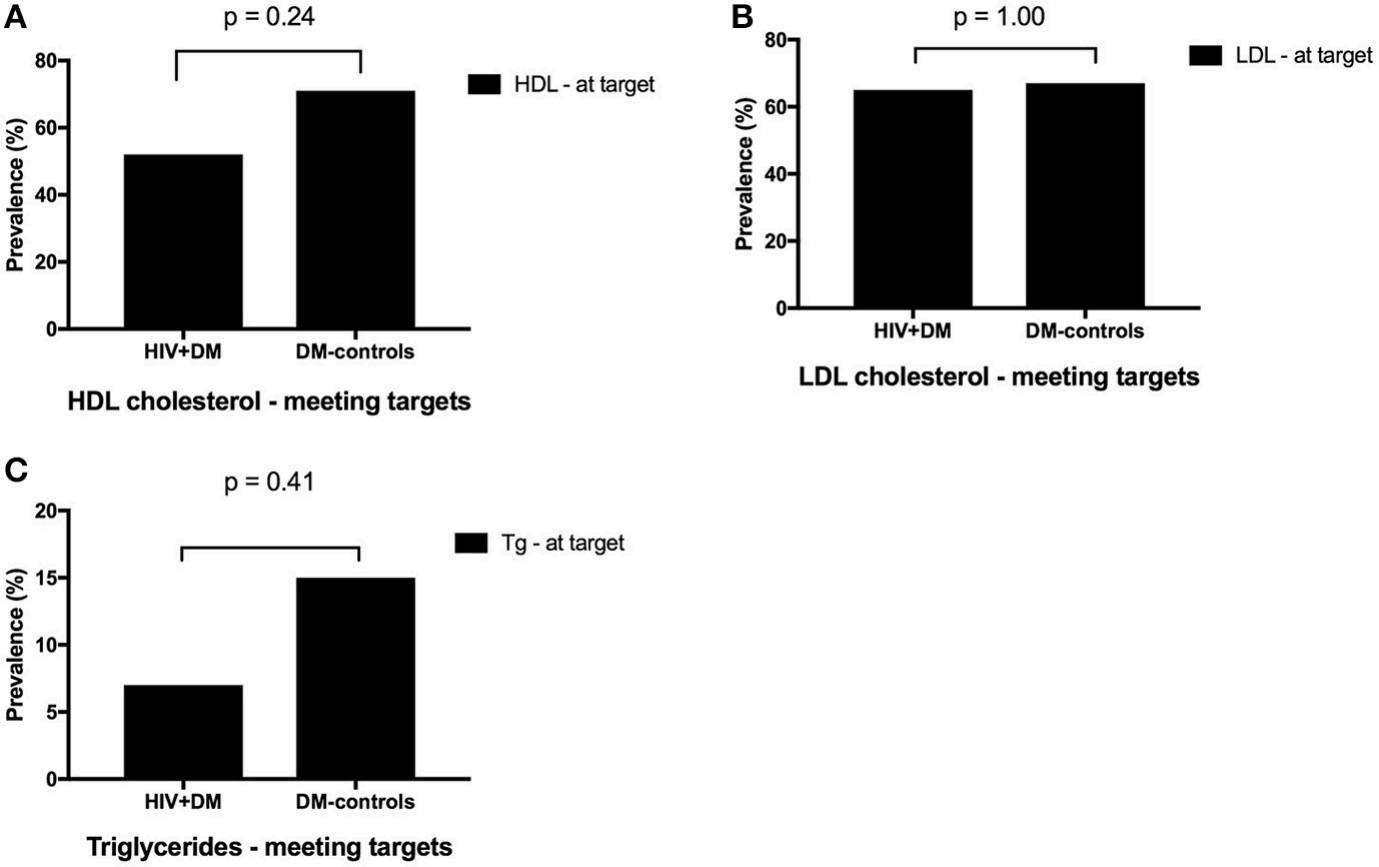
Proportion of clinic attendees meeting individualized ADA benchmarks for HDL cholesterol **(A)**, LDL cholesterol **(B)**, and triglycerides **(C)**.

**Figure 4:**
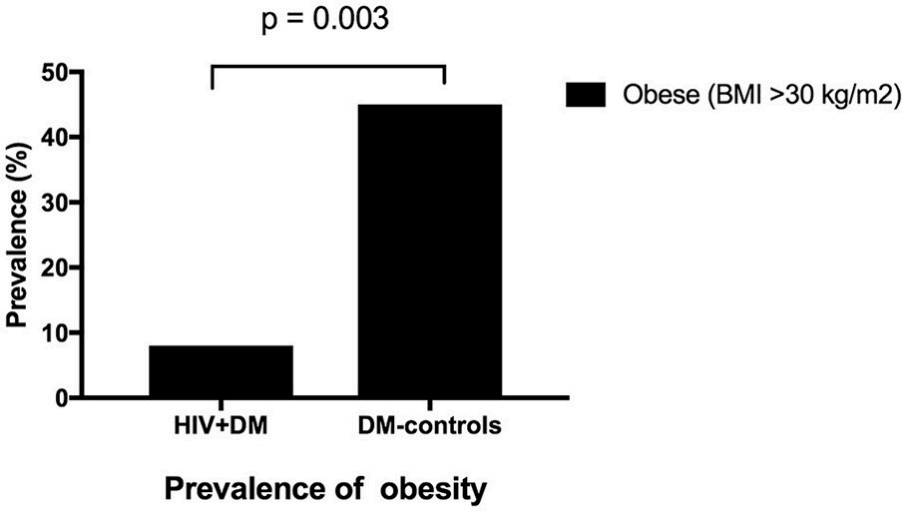
Prevalence of obesity in clinic attendees with both HIV-infection and diabetes vs. diabetes controls.

The mean HbA1c was 7.5 ± 1.5% for HIV+DM (T2DM *n* = 25, HbA1c 7.4 ± 1.4%; T1DM *n* = 4, 7.8 ± 2.2%) vs. 7.0 ± 1.4% for DM-controls (all had T2DM *p* = NS, Figure 1). The individualized HbA1c target was met less frequently in HIV+DM compared to DM-controls: 41 vs. 70%, respectively, *p* = 0.037 (Figure 2A). HIV+DM had significantly lower systolic BP than DM-controls (126 ± 10 mmHg vs.137 ± 17, respectively, *p* = 0.005) but similar diastolic blood pressures (Figure 1). Blood pressure targets were poorly met by both HIV+DM and DM-controls (43 vs. 23%, respectively, *p* = NS, Figure 2B). HIV+DM had higher total cholesterol vs. DM-controls (5.0 ± 1.8 vs. 4.0 ± 1.0 mmol/l, respectively, *p* = 0.017) and higher triglycerides (3.6 ± 4.2 vs. 1.8 ± 1.3 mmol, respectively, *p* = 0.043, Figure 1). The rate of lipid lowering medication prescription with statins was lower in HIV+DM (71%) compared to 86% in DM-controls. Fibrate use was similar between HIV+DM and DM-controls (18 vs. 10%, respectively, Table 1). HDL cholesterol targets were met in 52% of HIV+DM vs. 71% of DM-controls (*p* = NS, Figure 3A). LDL cholesterol targets were also sub-optimally met by both HIV+DM and DM-controls (65 vs. 67%, *p* = NS, Figure 3B). Triglyceride targets were very poorly met by both HIV+DM and DM-controls (7 vs. 15%, *p* = NS, Figure 3C). The prevalence of obesity (BMI ≥30.0 kg/m^2^) was significantly lower in HIV+DM compared to DM-controls (7.7 vs. 45%, respectively *p* = 0.003; Figure 4, Table 3).

Adherence to diabetes complication screening was below recommended benchmarks for both HIV+DM and DM-controls for annual foot examination (53 vs. 67%, respectively), biennial eye examination (83 vs. 77%, respectively; and annual urinary albumin measurement (77 vs. 67%, respectively). There were no significant differences observed in the delivery of microvascular complication screening between the two groups (Table 3).

HIV+DM were more likely to have 2 or more visits to the diabetes clinic in the study period (*p* = NS). Diabetes educator consultation was more frequent in the HIV+DM group (77%, *n* = 23) compared to DM-controls (47%, *n* = 14, *p* = 0.017). Dietician consultation was also more frequent in HIV+DM (57%, *n* = 17) compared to DM-controls (33%, *n* = 10, *p* = NS) but not significantly.

### Subgroup analysis of HIV+DM

In those with HIV+DM, subgroup analyzes were performed to examine for differences between those who did or did not meet individualized HbA1c targets (Table 4). Those meeting HbA1c targets were more likely to be healthy weight (BMI ≤24.9 kg/m^2^, 63 vs. 20%, respectively; *p* = 0.043) and less likely to be on insulin (25 vs. 47%, respectively, *p* = NS) or have HCV-co-infection (8 vs. 18%, respectively, *p* = NS) although the latter two were not statistically significant. They were similar to each other for all other variables (Table 4).

Additional sub-group analyzes were performed to examine for differences between HIV with T2DM vs. HIV with T1DM (Table 5). The T1DM group was younger than T2DM group (47.0 ± 9.2 vs. 58.2 ± 8.8 years, respectively, *p* = 0.027) and had longer duration of diabetes (22.5 ± 15 vs. 7.2 ± 5 years, *p* = NS). The mean BMI in T1DM group was lower than in the T2DM group (21.9 ± 3.0 vs. 26.2 ± 3.0 kg/m^2^ *p* = 0.007). The T1DM were more likely to be in the healthy weight range than T2DM group (BMI ≤ 24.9 kg/m^2^, 75% vs. 39%, respectively, *p* = NS). In the T1DM group, none were on metformin compared with 81% in T2DM group (*n* = 21, *p* = 0.05). All with T1DM were on insulin compared with 27% in T2DM group (*n* = 7, *p* = 0.012). The systolic blood pressure was lower in the T1DM group than T2DM group (118 ± 3.0 vs. 127 ± 9 mmHg, respectively, *p* = NS) as well as the diastolic blood pressure (68.8 ± 9 vs. 78.6 ± 7 mmHg, respectively, *p* = 0.018). They were similar to each other for all the other variables (Table 5).

Finally, subgroup analysis was done for patients with HIV+T2DM (excluding patients with T1DM) vs. controls. The mean BMI in HIV+T2DM was lower than in the DM-controls (26.2 ± 2.7 vs. 28.9 ± 6.3 kg/m^2^ *p* = NS) but it was not statistically significant.

### Diabetes complication prevalence

Table 6 shows the prevalence of diabetes macrovascular and microvascular complications where screening had occurred. There were 8 individuals (*n* = 4 in each group) with clinical macrovascular disease (i.e., history of cardiovascular disease and/or cerebrovascular disease); *n* = 2 with only cardiovascular disease (*n* = 1 in each group), *n* = 4 with only cerebrovascular disease (*n* = 2 in each group); *n* = 2 with both cardiovascular and cerebrovascular disease (*n* = 1 in each group). Overall, the prevalence of one or more complications was similar: macrovascular disease, 23% in HIV+DM and 23% in DM-controls; microvascular disease: 40 vs. 30%, respectively (*p* = NS).

## Discussion

Our single center retrospective study found that people with both HIV-infection and diabetes met the benchmarked standards of care for individualized HbA1c far less frequently compared to those with diabetes without HIV. According to the 2013 ADA guidelines that remain current and in clinical practice, the target HbA1c should be based on duration of diabetes, age/life expectancy, known cardiovascular disease, advanced micro-vascular complications, hypoglycaemic unawareness or comorbid conditions (8).

This is the first study to compare HbA1c targets between people with both HIV-infection and diabetes to matched individuals attending the same clinic. Further, this is also the first study to examine individualized HbA1c target achievement. Prior studies from two American groups showed that a target of HbA1c ≤7.0% (53 mmol/mol) was met in 54–57% of patients with HIV+DM (6, 12); both studies lacked control groups. The U.S. National Health and Nutrition Examination 2007–2010 survey has shown similar target rates achieved for USA adults with diabetes (13–15). Similar rates were shown in an uncontrolled study from the Netherlands: 54% of patients with HIV+DM achieved target HbA1c ≤7% (53 mmol/mol) (16). Prior studies have documented that inadequate glycaemic control was associated with more recent HIV diagnosis, requiring insulin or other oral hypoglycaemic agents and higher triglyceride levels (6).

It is noteworthy that only 70% of the DM-control group achieved target glycaemic control. This may reflect the more complex patient cohort attending a tertiary center ambulatory clinic, with diabetes onset at an earlier age: our mean age was 57 years and known duration of diabetes was 10 years; one quarter had one or more macro-vascular complication and one third one or more micro-vascular complication. Nevertheless, even with this more complex control cohort, our findings suggest disparities in glycaemic management in people with HIV+DM vs. DM-controls exist.

Blood pressure targets were met sub-optimally in both groups. Only 43% of patients with HIV+DM met the target, despite 86% of patients prescribed antihypertensive therapy. While this was higher than in the DM+control group where 23% met the target, both were below the standard of care. This is similar to published data, that show that 42–56% of patients with HIV+DM achieved the target <130/80 mmHg at HIV-specialist clinics (6, 12) compared to 51% of DM-controls at general medicine clinics (15). While white coat hypertension cannot be excluded and may contribute to clinical inertia, these findings suggest that despite blood pressure monitoring and medication prescription, there is insufficient clinical action in the face of finding ongoing hypertension in both groups. Interestingly, the systolic and diastolic blood pressures were lower in the HIV-infected individuals with T1DM than T2DM. In T1DM, arterial hypertension is associated with microalbuminuria and is a consequence of renal disease and poor glycaemic control with onset usually years after diagnosis in contrast to arterial hypertension in T2DM, that is part of the metabolic syndrome and is associated with dyslipidaemia, central obesity, insulin resistance and is often present at diagnosis (17).

HDL cholesterol targets were met at similar rates in HIV+DM vs. DM-controls; similar data were reported by Adeyami et al. (*n* = 216 HIV-infected diabetic patients) (12). LDL cholesterol targets were also similarly met in HIV+DM and DM-controls, similar to published data from HIV-specialist clinics which have reported 40–66% achieving LDL targets (6, 12). In the general population, 56% of patients are meeting the LDL cholesterol target (15). The triglyceride target was poorly met by both HIV+DM and DM-controls, lower than reported by Satlin et al. (6) (31%). The high rates of refractory dyslipidaemia in people with HIV+DM may be related to medications used to suppress HIV replication. For example, protease inhibitor medications which are often used in combined antiretroviral therapy are associated with mixed hyperlipidemia and insulin resistance (18–21), as are thymidine analoge nucleoside reverse transcriptase inhibitors (22). Of note, non-nucleoside reverse transcriptase inhibitors efavirenz and nevirapine increase HDL while fusion inhibitors and integrase strand transfer inhibitors have neutral effects on the lipids demonstrating cART plays a large role in lipid metabolism and choice of therapy can effect target lipid outcomes (23). Treatment options for dyslipidaemia include changing cART to a more lipid-neutral regimen and using medical therapy (24). Statins are generally first line therapy due to their ability to reduce cardiovascular morbidity and mortality in primary and secondary prevention and ezetimibe can be used in statin-intolerant individuals or as add on therapy (23, 24). For patients with isolated hypertriglyceridaemia, omega 3 fatty acid supplementation has been shown to be effective as shown in a meta-analysis including 20 randomized controlled studies (*n* = 1,209 participants) that demonstrated individuals on supplements had triglyceride levels significantly decreased compared to controls (25). Fenofibrates and omega 3 fatty acid supplementation can be used alone or in combination, however care must be taken with fenofibrates if on statin therapy due to increased risk of skeletal toxicity (23, 24). Proprotein convertase subtilisin/kexin type 9 (PCSK9) inhibitors are a novel class of drugs that block the activity of PCSK9, a proprotein convertase that has a role in breaking down LDL receptors in the liver (24, 26). Mutations in the PCSK9 gene are responsible for familial hypercholesterolemia that is a result of reduced LDL receptors on hepatocytes and reduced ability to remove LDL cholesterol from plasma (26).

By reducing the breakdown of LDL receptors, they increase the clearance of LDL cholesterol with overall reduction in LDL cholesterol (24, 26). They were not available during the study period.

In our study HIV+DM patients were less likely to be overweight or obese than DM-controls, and this was even more pronounced for HIV-infected with T1DM vs. HIV-infected with T2DM. Despite this, the prevalence of cardiovascular risk factors and metabolic syndrome phenotypes were still highly prevalent. Joy et al. (27) reported that for any given BMI, people with HIV-infection have 1.1 kg less limb fat than controls. Additionally, among people with HIV-infection with healthy weight or overweight group (BMI 18.5–29.9 kg/m^2^), visceral adipose tissue mass is higher and subcutaneous adipose tissue lower compared to controls without HIV infection (27). Further, a prospective study found high rates of incident glucose disorders and incident diabetes in men living with HIV-infection over a mean follow-up of 11 years (28). Importantly, most of the men were healthy weight. A modest, early gain in visceral fat was associated with a 3-fold increased risk of an incident glucose disorder (28). Therefore, a lower BMI in people with HIV-infection carries an increased risk of cardiometabolic complications, contributed to (at least in part) by greater visceral adiposity (27). It is important to recall that anti-retroviral agents have been implicated in the pathogenesis of premature ischaemic heart disease in this group (29). People living with treated HIV-infection are at higher risk of premature diabetes (28) and ischaemic heart disease (30, 31). Diabetes increases the risk of cardiovascular disease by more than 2-fold (32) and HIV is also associated with increased cardiovascular risk, with studies showing almost 2-fold increased risk of myocardial infarction after adjusting for age, gender, race hypertension, diabetes, and dyslipidaemia (33, 34). The large Data Collection on Adverse Events on Anti-Retroviral Drugs study estimated 16% risk per annum of a myocardial infarct after starting on anti-retroviral therapy (29). The high prevalence of ongoing hypertension and hyperlipidaemia in our setting suggests treatment disparities and physician inertia, but may also reflect the refractoriness of cardiovascular risk factors to standard therapies, particularly to standard statin therapy. Further, it needs to be recalled that various statin medications are metabolized by the cytochrome P450 system, which is inhibited by some antiretroviral medications, particularly protease inhibitors, which may limit use of the most potent statins (35). Concern for drug-drug interactions may be a factor contributing to what appears to be clinical inertia in bringing lipids to targets in people with HIV+DM.

Metabolic syndrome is associated with abdominal obesity, dyslipidaemia, inflammation, insulin resistance or diabetes, and increases the risk of cardiovascular disease (36). The cardiovascular risk is greatest when both metabolic syndrome and diabetes are present with prevalence of cardiovascular heart disease in 19.2% of individuals ≥50 years of age compared to 13.9% in those with metabolic syndrome without diabetes and 7.5% in individuals with diabetes without metabolic syndrome, based on the data from the Third National Health and Nutrition Examination Survey (37, 38). We have previously shown that lean men with treated HIV-infection have a pro-inflammatory profile equivalent to individuals with insulin-resistant obesity and these factors contribute to accelerated diabetes and cardiovascular risk (34). In our study we have shown HIV-infected men have high rates of uncontrolled lipids, blood pressure and glucose *despite* being healthier weight than controls and this is associated with high cardiac risk.

There is a large amount of evidence demonstrating that a healthy diet and regular physical activity reduce cardiovascular risk (24, 39). The Mediterranean diet, with emphasis on consumption of fish 2–3 × per week, increased consumption of fresh fruit, vegetables, legumes, and limited consumption of animal saturated fat, has been shown to be protective against cardiovascular disease and mortality as demonstrated in the PrediMed trial with 7,447 participants (24, 39). There is also data demonstrating a hypocaloric diet low in saturated fats reduces triglyceride and LDL cholesterol levels and reduces risk of cardiovascular and atherosclerotic diseases (23). Regular physical activities improve muscle strength, endurance and cardiovascular fitness and in turn also increase HDL cholesterol as shown by Kraus et al. in randomized controlled study (*n* = 111) (40) and by Kodama et al. in a meta-analysis of all studies (*n* = 25 studies) evaluating aerobic exercise and the effects on HDL cholesterol (41). In the STRRIDE study, a randomized controlled study with 120 participants, exercise was associated with reduced abdominal, waist and hip circumferences as well as weight in dose-dependent fashion with greater reduction in high-intensity exercise group compared to low-intensity exercise group (42).

Our complication screening was similar between the HIV+DM and DM-control men and higher than previously reported. Fifty three percent of our HIV+DM men had an annual foot examination compared to 18% reported by Adeyemi et al. (12), 83% had a biennial retinal examination compared with 47% reported by Satlin et al. (6) and 77% had an annual urine albumin creatinine measured, compared to 62% reported by Adeyemi et al. (12). Our high biennial retinal examination rates are likely due to access to a retinal camera available at our diabetes center. The prevalence of macro- and micro-vascular complications was similar between the HIV+DM vs. DM-controls groups with 23% in each group having one or more macro-vascular complications (*p* = NS) and 40 vs. 30%, respectively (*p* = NS) having one or more micro-vascular complications. These rates suggest a complex group of patients with more disease burden.

Excess weight is a known contributing factor to progression of diabetes and deteriorated glycaemic control (36). In people living with treated HIV infection, particularly those with lipodystrophy or central obesity, addressing excess weight with individualized lifestyle interventions focusing on dietary intake and physical activity will assist in addressing the harmful effects of excess weight. Pharmacotherapies that can also assist in both weight management include metformin and the glucagon peptide-1 agonists exenatide and liraglutide (43). Bariatric surgery is also an option for the obese who do not respond to lifestyle interventions and pharmacotherapy (8). HCV co-infection is associated with peripheral insulin resistance and poor glucose control in diabetes (44). Current pharmacotherapy for HCV using Sofosbuvir and Velpatasvir as shown high rates of virus eradication (45) and improved glucose control (46, 47). Therefore, people with HCV co-infection should be offered access to HCV eradication.

Limitations of our study were small study size and retrospective study design. Our cohort were predominantly inner city-dwelling men who have sex with men; the majority received cART; therefore our findings may not be generalisable to treated HIV-infection and intravenous drug use, female sex, untreated HIV-infection or resource-poor settings where there is limited access to HIV-specialist clinics and resources.

In summary, we found that glycaemic control, blood pressure, and lipid targets in individuals with HIV+DM were below the standard of care in a large inner city tertiary hospital with a long tradition for treatment of people with HIV-infection. Rates of blood pressure control and triglycerides were similarly poor for controls with DM. Our findings suggest there may be treatment disparities (related to clinical differences or clinical inertia) in delivering target glycaemic and lipid control to people with HIV+DM. Complication screening was higher than reported in prior studies. As HIV-infected patients are enjoying near-normal life expectancy and at greater risk of diabetes-related complications (2) and cardiovascular disease, it is critical to achieve benchmarked standards of care. Increased diabetologist education, internal audits of glycaemic, lipid and clinical targets and adherence to complication screening may benefit. Greater emphasis on diet and exercise in conjunction with medical therapy may reduce the metabolic and cardiovascular manifestations. Further, embedding a specialized diabetologist within HIV specialist services may be an opportunity to ensure the standards of care are met for people living with both HIV+DM.

## Ethics statement

We received Ethics approval for this study through St Vincent's hospital Ethics committee. HREC reference number LNR/14/SVH/149

## Author contributions

All authors designed the study. MF-L collected the data and wrote the first draft of the manuscript. Both KT and KS contributed equally to reviewing the manuscript, tables and figures and making revisions to it.

### Conflict of interest statement

The authors declare that the research was conducted in the absence of any commercial or financial relationships that could be construed as a potential conflict of interest.
